# COMPARATIVE PERFORMANCE OF FOUR CLAIMS-BASED FRAILTY MEASURES IN THE UNITED STATES MEDICARE DATA

**DOI:** 10.1093/geroni/igz038.2526

**Published:** 2019-11-08

**Authors:** Dae H Kim, Elisabetta Patorno, Ajinkya Pawar, Hemin Lee, Sebastian Schneeweiss, Robert Glynn

**Affiliations:** 1 Hebrew SeniorLife, Boston, Massachusetts, United States; 2 Brigham and Women’s Hospital and Harvard Medical School, Boston, Massachusetts, United States; 3 Brigham and Women’s Hospital, Boston, Massachusetts, United States

## Abstract

Background: There has been increasing effort to measure frailty in the United States Medicare data. The performance of claims-based frailty measures has not been compared. Methods: This retrospective cohort study included 2,326 community-dwelling Medicare beneficiaries who participated in the 2008 assessment of the Health and Retirement Study. The claims-based frailty measures developed by Davidoff, Faurot, Segal, and Kim were compared against clinical measures of frailty (gait speed, grip strength) using correlation coefficients and health outcomes (e.g., mortality, hospitalization, activities-of-daily-living disabilities) over 2 years using C-statistics. Results: The Davidoff, Faurot, Segal, and Kim indices were negatively correlated with gait speed (-0.19, -0.33, -0.37, and -0.37, respectively), but age and sex adjustment variably attenuated the correlation to -0.17, -0.22, -0.18, and -0.33, respectively. The corresponding correlation coefficients with grip strength were -0.17, -0.27, -0.35, and -0.24, which attenuated to -0.09, -0.14, -0.05, and -0.23 after age and sex adjustment, respectively. The models that included age, sex, and each of Davidoff, Faurot, Segal, and Kim indices showed C-statistics of 0.67, 0.71, 0.71, 0.75 for mortality (versus C-statistic for age and sex: 0.66); 0.59, 0.64, 0.63, 0.70 for hospitalization (versus C-statistic for age and sex: 0.58); and 0.64, 0.63, 0.63, 0.70 for activities-of-daily-living disabilities (versus C-statistic for age and sex: 0.61), respectively. Conclusions: The choice of a claims-based frailty measure results in a meaningful variation in the identification of frail older adults at high risk for adverse health outcomes. Claims-based frailty measures that included demographic variables offer limited risk adjustment beyond age and sex.

